# Protocol for isolation of nuclei from murine cardiac tissue for single-nucleus multiomic sequencing

**DOI:** 10.1016/j.xpro.2026.104615

**Published:** 2026-06-06

**Authors:** Ioanni Veras, Olav Søvik Eken, Finn Olav Levy, Arne Olav Melleby, Jan Magnus Aronsen

**Affiliations:** 1 Department of Molecular Medicine, Institute of Basic Medical Sciences, University of Oslo, Oslo, Norway; 2 Department of Pharmacology, Institute of Clinical Medicine, University of Oslo, Oslo, Norway; 3 Department of Pharmacology, Division of Laboratory Medicine, Oslo University Hospital, Oslo, Norway

**Keywords:** Single Cell, Genomics, Molecular Biology

## Abstract

Pathological cardiac remodeling involves cell-type-specific regulatory changes that require integrative analysis across all cardiac cell populations. Here, we present a protocol for isolating single nuclei from fresh-frozen murine cardiac ventricular tissue to enable the integrated analysis of gene expression and chromatin accessibility. We describe steps for mechanical homogenization, sequential filtration, sucrose cushion purification, and fluorescence-activated nuclei sorting (FANS). This protocol enables multiomic analysis across various cardiac cell types and supports epigenomic studies of gene regulation.

## Highlights

• Nuclei isolation from snap-frozen murine left ventricle tissue
• Debris removal and FANS purification of cardiac nuclei
• Integrated analysis of snRNA-seq and snATAC-seq from the same nuclei
• Mapping of transcription factor binding motifs in murine cardiac nuclei

## Abstract (editor highlights)

Publisher’s note: Undertaking any experimental protocol requires adherence to local institutional guidelines for laboratory safety and ethics.

## Graphical abstract

**Figure:**
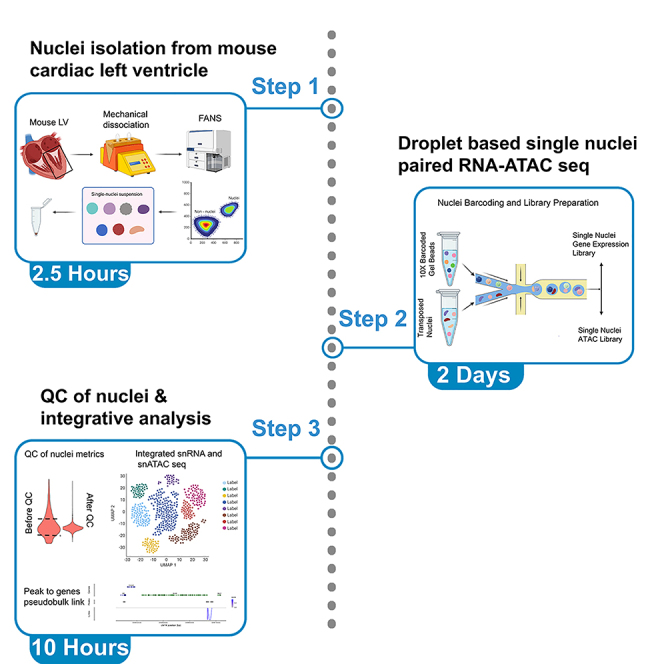

## Before you begin

High-throughput transcriptomic and epigenomic profiling at single-cell and single-nucleus resolution has become a powerful approach for advanced medical research, particularly through droplet-based platforms and paired RNA–chromatin accessibility workflows.^1^^,^^2^^,^^3^^,^^4^^,^^5^^,^^6^^,^^7^ In the heart, these approaches are especially valuable because cardiac tissue contains diverse cell populations and is difficult to dissociate at the whole-cell level owing to its fibrous architecture, large cardiomyocyte size, and structural fragility.^8^^,^^9^^,^^10^^,^^11^ Single-nucleus approaches are therefore particularly well suited for cardiac tissue, including frozen samples and challenging clinical or postmortem material.^4^^,^^5^^,^^6^^,^^8^^,^^11^^,^^12^^,^^13^ The protocol described here details the isolation of nuclei from one whole snap-frozen murine left ventricle (∼20–40 mg) for downstream single nucleus paired chromatin accessibility + gene expression profiling using the 10x Genomics Chromium platform.^14^ All the mouse experiments were performed in agreement with the Guide for the Care and Use of Laboratory Animals and approved by the Norwegian National Animal Research Committee Reporting of experimental procedures and results followed the ARRIVE guidelines.^15^^,^^16^ All experiments therefore conformed to the relevant institutional and national regulatory standards. Use of this protocol on laboratory animal or patient tissue must obtain approval from the appropriate ethics and regulatory authorities at their own institutions and ensure that all experiments are performed in accordance with applicable national and institutional guidelines.

**Figure 1:**
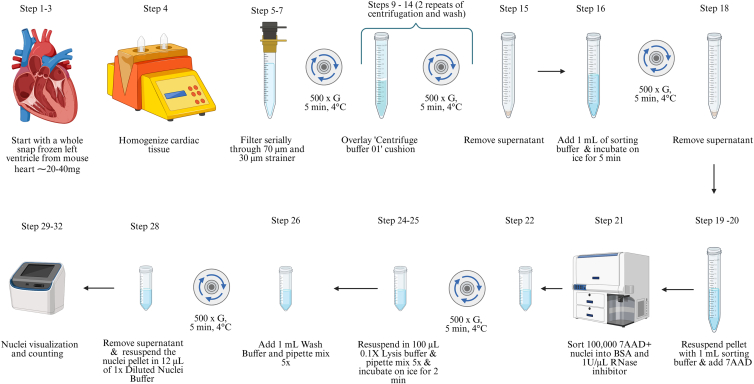
Schematic representation of the proposed workflow for isolation of nuclei from frozen murine ventricular tissue for single-nucleus multiomic sequencing Frozen cardiac tissue is homogenized, filtered, and subjected to cushion centrifugation to enrich for cardiac nuclei. Further debris and aggregates removal is based on FANS yielding nuclei suitable for single-nucleus multiomic sequencing.

This protocol has been validated in snap-frozen murine left ventricular tissue derived from both healthy and fibrotic hearts. Moreover it was optimized for samples in which minimization of debris carryover and compatibility with paired transcriptomic and chromatin-accessibility profiling are critical. Every step of this protocol has been optimized for one whole murine cardiac left ventricle(∼20–40 mg). If more tissue is available, it is preferable to process a portion of the sample that is close to this recommended input range, rather than substantially increasing the amount of starting material for a single preparation. Excessive tissue input may increase debris carryover, nuclei aggregation, and sorting burden. In the manuscript, the nuclei preparation is intended recovery of approximately 15,000 nuclei per sample, and final nuclei suspensions are prepared at approximately 4,000–8,000 nuclei/μL. An overview of the workflow is shown in Figure 1.

### Innovation

**Figure 2:**
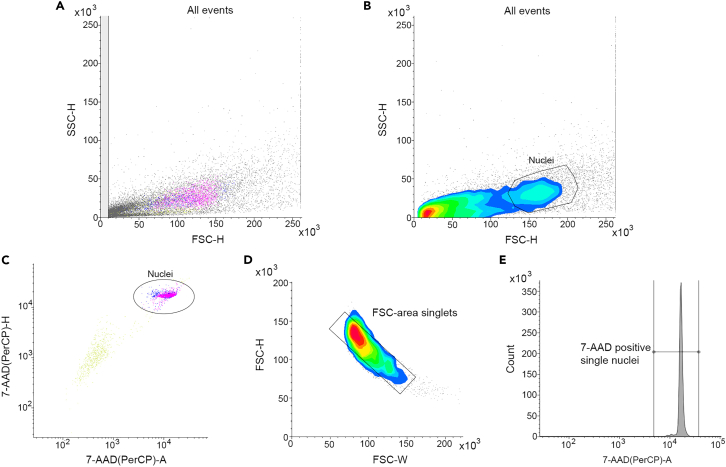
Flow Activated Nuclei Sorting (FANS) workflow for capturing intact cardiac nuclei populations and removing all unstained debris (A and B) Forward scatter (FSC) and side scatter (SSC) parameters are used to identify the nuclei populations based on their size. (C) Fluorescent gating with 7AAD distinguishes stained nuclei (7AAD-positive) from non-nuclear debri (7AAD-negative). (D) A third gate, based on FSC width/height, discriminates single nuclei from aggregates. (E) Histogram of sorted nuclei based on the 7AAD positive signal.

**Figure 3:**
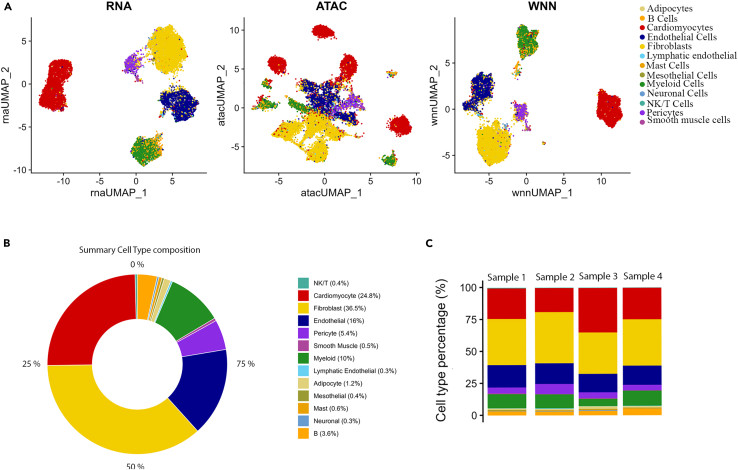
UMAP visualization of snRNA-seq, snATAC-seq, and WNN-integrated snMultiome clustering and cell type composition across the dataset and individual samples (A) UMAP projections based on snRNA-seq (left), snATAC-seq (middle), and integrated snMultiome/WNN analysis (right), showing the cardiac cell populations. (B) Overall cell type composition across all nuclei included in the analysis. (C) Cell type composition shown separately for each sample.

The protocol presented here provides a standardized framework for isolating single-nuclei solutions from snap-frozen murine cardiac left ventricular tissue for paired single-nucleus RNA-seq and Assay for Transposase-Accessible Chromatin (ATAC)-seq. The individual experimental steps build on established nuclei-isolation procedures but were specifically optimized to address the technical challenges posed by fibrous cardiac tissue, including low yield, debris carryover, and nuclear aggregation. The innovative potential of this protocol lies in its integral combination of standardized gentleMACS-based mechanical dissociation, serial filtration, repeated sucrose-cushion purification, and fluorescence-activated nuclei sorting (FANS) using 7-AAD staining (Figure 2) to generate concentrated and quantifiable single-nuclei suspensions compatible with the 10x Genomics Chromium Single Cell Multiome platform. Compared with more operator-dependent manual homogenization workflows, this protocol is designed to improve reproducibility while preserving nuclear yield. Using this approach, the authors recovered nuclei across all the major cardiac cell populations (Figure 3) and achieved robust profiling of both transcriptional state and chromatin accessibility from the same sample (Figure 3A). We further propose that this workflow may serve as a template for single-nucleus multiomic analysis in other difficult-to-dissociate or frozen tissues, although broader validation beyond murine ventricular tissue remains to be established.

### Institutional permissions

Mouse experiments were performed in agreement with the Guide for the Care and Use of Laboratory Animals (NIH publication No. 85-23, revised 2011, US) and approved by the Food Safety Authority (approval #18679, #23193 and #30837). Reporting of experimental procedures and results followed the ARRIVE guidelines.^15^^,^^16^

## Key resources table

**Table:** 

REAGENT or RESOURCE	SOURCE	IDENTIFIER
**Antibodies**		

Anti – Lamin B1; 1:500	Abcam	ab16048
Goat anti-rabbit IgG (H+L) Alexa Fluor 488; 1:500	Thermo Fisher Scientific	A11034

**Chemicals, peptides, and recombinant proteins**		

CaCl2	Sigma	C1016
MgAc (1M)	Sigma	63052
EDTA	Sigma	E9884
EGTA	Sigma	E3889
Tris-HCL	Sigma	T3253
NAF	Sigma	201154
Sodium Butyrate	Sigma	B5887
DTT (1M)	Sigma	646563
10X PBS Buffer pH 7.4	Thermo Fisher Scientific	AM9624
MACS BSA stock solution	Miltenyi Biotec	130-091-376
Digitonin (5%)	Thermo Fisher Scientific	BN2006
Nuclei Buffer (20X)	10xGenomics	2000153/2000207
7-AAD Ready Made Solution	Sigma	SML1633
NaCl (5M)	Sigma	59222C
MgCl2 (1M)	Sigma	M1028
Protector RNase Inhibitor	Sigma	3335402001
cOmplete™, Mini, EDTA-free Protease Inhibitor Cocktail	Sigma	11836170001
Actinomycin D	Sigma	A4262
Trypan Blue solution	Sigma	T8154
Sodium Orthovanadate, Activated	Sigma	5086050004
Triton X-100	Sigma	T8787
Igepal CA-630	Sigma	I8896
Tween-20	Sigma	P1379
Nuclease-free water	Thermo Fisher Scientific	AM9932
Sucrose	Sigma	S0389
RNase*Zap*™ RNase Decontamination Solution	Thermo Fisher Scientific	AM9780

**Critical commercial assays**		

Chromium Next GEM Single Cell Multiome ATAC + gene expression reagent bundle, 16 rxns	10x Genomics	1000283
Chromium Next GEM Chip J Single Cell Kit, 48 rxns	10x Genomics	1000234
Dual index kit TT set A, 96 rxns	10x Genomics	1000215

**Experimental models: Organisms/strains**		

C57BL/6JRj 9 weeks old male mice	Janvier Labs, Le Genest-Saint-Isle, France	–

**Software and algorithms**		

Cell Ranger ARC version 2.0.2	10x Genomics	https://www.10xgenomics.com/support/software/cell-ranger-arc/latest
R version 4.3.3	R Project	https://www.r-project.org/
Seurat	v5.2.0	https://satijalab.org/seurat/index.htmL
Signac	v1.16.0	https://github.com/stuart-lab/signac
Azimuth	v0.5.0	https://github.com/satijalab/azimuth
chromVAR	v1.24.0	https://github.com/GreenleafLab/chromVAR
JASPAR2020	v0.99.10	https://jaspar.elixir.no/
EnsDb.Mmusculus.v79	v2.99.0	https://www.bioconductor.org/packages/EnsDb.Mmusculus.v79/
Dplyr	v 1.2.0	https://cran.r-project.org/package=dplyr
scDblFinder	v1.16.0	https://bioconductor.org/packages/release/bioc/htmL/scDblFinder.htmL
patchwork	v 1.3.2	https://patchwork.data-imaginist.com/
ggplot2	v4.0.2	https://cran.r-project.org/package=ggplot2
SeuratData	v0.2.2.9001	https://github.com/satijalab/seurat-data
chromVAR	v1.24.0	https://bioconductor.org/packages/release/bioc/htmL/chromVAR.htmL
TFBSTools	V1.40.0	https://bioconductor.org/packages/TFBSTools/
motifmatchr	V1.24.0	https://bioconductor.org/packages/TFBSTools/
harmony	V1.2.0	https://github.com/immunogenomics/harmony
BSgenome.Mmusculus.UCSC.mm10	v1.4.3	https://bioconductor.statistik.tu-dortmund.de/packages/3.12/data/annotation/htmL/BSgenome.Mmusculus.UCSC.mm10.htmL

**Other**		

DNA LoBind tubes, 0.5 mL	Eppendorf	022431005
DNA LoBind tubes, 1.5 mL	Eppendorf	022431021
DNA LoBind tubes, 2.0 mL	Eppendorf	022431102
DNA LoBind tubes, 5.0 mL	Eppendorf	0030108310
Protein LoBind tubes, 15 mL	Eppendorf	0030122216
Protein LoBind tubes, 50 mL	Eppendorf	0030122240
Countess 3 FL automated cell counter	Thermo Fisher Scientific	AMQAX2000
gentleMACS™ Dissociator	Miltenyi Biotec	130-093-235
gentleMACS™ C Tubes	Miltenyi Biotec	130-093-237
MACS® SmartStrainers (30 μm)	Miltenyi Biotec	130-110-915
MACS® SmartStrainers (70 μm)	Miltenyi Biotec	130-098-462
Swinging bucket centrifuge	Thermo Fisher Scientific	75016033
High Sensitivity D1000 ScreenTape	Agilent	5067–5584
High Sensitivity D1000 Ladder	Agilent	5067–5587
2100 Bioanalyzer	Agilent	G2939A
High Sensitivity DNA Kit	Agilent	5067–4626
NovaSeq 6000	Illumina	N/A

## Materials and equipment

**Table:** 10% Triton X-100 buffer

Component (Storage)	Stock	Final	50 mL
Triton X-100 (RT)	100%	10%	5
Nuclease-free Water (RT)	-	-	45

Prepare in advance and stored up to six months at 4°C

**Table:** 10% IGEPAL buffer

Component (Storage)	Stock	Final	50 mL
IGEPAL CA-630 (RT)	100%	10%	5
Nuclease-free Water (RT)	-	-	45

Prepare in advance and stored up to six months at 4°C

**Table:** 10% Tween-20 buffer

Component (Storage)	Stock	Final	50 mL
Tween-20 (RT)	100%	10%	5
Nuclease-free Water (RT)	-	-	45

Prepare in advance and stored up to six months at 4°C

**Table:** 25x cOmplete Protease Inhibitor Cocktail buffer

Component (Storage)	Stock	Final	2 mL
cOmplete Protease Inhibitor Cocktail (4°C)	1 tablet	10%	1 tablet
Nuclease-free Water (RT)	-	-	2 mL

Prepare in advance and stored up to six months at 4°C

**Table:** Homogenization buffer 00

Component (Storage)	Amount	Final concentration (mM)
CaCl2 (RT)	416.17 mg	5
MgAc (1M) (RT)	2.250 mL	3
EDTA (RT)	438.36 mg	2
EGTA (RT)	142.52 mg	0.5
Tris-HCL (RT)	1182 mg	10
NAF (RT)	157 mg	5
Sodium Butyrate (RT)	330.24 mg	5
Nuclease-free Water (RT)	600 mL	-

Adjust to pH 8, prepare in advance and store up to six months at 4°C. Filter it through a 0.2μm filter once per week.

**Table:** Homogenization buffer 01

Component(storage)	Volume	Final
Homogenization buffer 00 (4°C)	40 mL	-
Actinomycin D (10 mg/mL) (−20°C)	5 μL	1 μg/mL
Sodium Orthovanadate Activated (−80°C) (53mM)	833 μL	1 mM
Nuclease-free Water (RT)	4.167 mL	-

Prepare the same day and store on wet ice or at 4°C before use.

**Table:** Homogenization buffer 02

Component(storage)	Volume	Final
Homogenization buffer 01 (4°C)	9 mL	-
25x cOmplete Protease Inhibitor Cocktail buffer (4°C)	400 μL	0.5 μL/mL
RNase inhibitor 40U/μl (−20°C)	250 μL	1 U/μL
Nuclease-free Water (RT)	600 μL	-

Prepare the same day and store on wet ice or at 4°C before use.

**Table:** Homogenization buffer 03

Component(storage)	Volume	Final
Homogenization buffer 01(4°C)	9 mL	-
25x Protease inhibitor buffer (4°C)	400 μL	0.5 μl/mL
10% Triton X-100 (4°C)	400 μL	0.4 %
RNase inhibitor 40U/μl (−18°C)	250 μL	1 U/μL
Nuclease-free Water (RT)	200 μL	-

Prepare the same day and store on wet ice or at 4°C before use.

**Table:** Homogenization buffer 04

Component(storage)	Volume	Final
Homogenization buffer 01(4°C)	9 mL	-
25x Protease inhibitor buffer (4°C)	400 μL	0.5 μL/mL
10% Triton X-100 (4°C)	200 μL	0.2 %
RNase inhibitor 40U/μl (−18°C)	250 μL	1 U/μL
Nuclease-free Water (RT)	400 μL	-

Prepare the same day and store on wet ice or at 4°C before use.

**Table:** Centrifuge buffer 00

Component(storage)	Volume	Final
Sucrose (RT)	17115 mg	1 M
MgAc (1M) (RT)	150 μL	3 mM
Tris-HCL (RT)	78.8 mg	10 mM
Nuclease-free Water (RT)	50 mL	-

Adjust to pH 8, prepare in advance and store up to one week at 4°C.

**Table:** Centrifuge buffer 01

Component(storage)	Volume	Final
Centrifuge buffer 00 (4°C)	19.414 mL	-
Actinomycin D (10 mg/mL) (−20°C)	1.5 μL	1 μg/mL
BSA (4°C)	300 μL	1%
25x Protease inhibitor buffer (4°C)	960 μL	0.5 μL/mL
DTT (RT)	24 μL	1 mM
RNase inhibitor 40U/μl (−20°C)	600 μL	1 U/μL

Prepare the same day and store on wet ice or at 4°C before use.

**Table:** Sorting buffera

Component(storage)	Volume	Final
10x PBS (RT)	1050 μL	1×
BSA (4°C)	1200 μL	1%
RNase inhibitor 40U/μl (−20°C)	300 μL	1 U/μL
DTT (RT)	12 μL	1 mM
Nuclease-free Water (RT)	9450 mL	-

Prepare the same day and store on wet ice or at 4°C before use.

**Table:** 1x Lysis Buffera

Component (storage)	Stock	Final	2 mL
Tris-HCL pH 7.4 (RT)	1 M	10 mM	20 μL
NaCl (RT)	5 M	10 mM	4 μL
MgCl2 (RT)	1 M	3 mM	6 μL
Tween-20 (RT)	10%	0,1%	20 μL
IGEPAL CA-630 (RT)	10%	0,1%	20 μL
Digitonin (Incubate at 65°C to dissolve precipitate before use) (4°C)	5%	0,01%	4 μL
BSA (4°C)	10%	1%	200 μL
DTT (RT)	1000 mM	1 mM	2 μL
RNase inhibitor 40U/μL (−20°C)	40 U/μL	1 U/μL	50 μL
Nuclease-free Water (RT)	-	-	1.67 mL

Prepare the same day and store on wet ice or at 4°C before use.

**Table:** Lysis Dilution Buffera

Component (storage)	Stock	Final	2 mL
Tris-HCL pH 7.4 (RT)	1 M	10 mM	20 μL
NaCl (RT)	5 M	10 mM	4 μL
MgCl2 (RT)	1 M	3 mM	6 μL
BSA (4°C)	10%	1%	200 μL
DTT (RT)	1000 mM	1 mM	2 μL
RNase inhibitor 40U/μL (−20°C)	40 U/μL	1 U/μL	50 μL
Nuclease-free Water (RT)	-	-	1.718 mL

Prepare the same day and store on wet ice or at 4°C before use.

**Table:** Diluted Nuclei Buffera

Component (storage)	Stock	Final	2 mL
Nuclei Buffer 20x (−20°C; provided by 10× Genomics kit)	20×	1×	50 μL
DTT (RT)	1000 mM	1 mM	2 μL
RNase inhibitor 40U/μL (−20°C)	40U/μL	1U/μL	25 μL
Nuclease-free Water (RT)	-	-	924 μL

Prepare the same day and store on wet ice or at 4°C before use.

**Table:** 0.1x Lysis Buffera

Component (storage)	Stock	Final	2 mL
1x Lysis Buffer (ice)	1×	0.1×	200 μL
Lysis Dilution Buffer (ice)	-	-	1.8 mL

Prepare the same day and store on wet ice or at 4°C before use.

**Table:** Wash Buffer^a^

Component (storage)	Stock	Final	2 mL
Tris-HCL (pH 7.4) (RT)	1 M	10 mM	20 μL
NaCl (RT)	5 M	10 mM	4 μL
MgCl2 (RT)	1 M	3 mM	6 μL
BSA (4°C)	10%	1%	200 μL
Tween-20 (4°C)	10%	0.1%	20 μLK
DTT (RT)	1000 mM	1 mM	2 μL
RNase inhibitor 40U/μL (−20°C)	40 U/μL	1 U/μL	50 μL
Nuclease-free Water (RT)	-	-	1.718 mL

Prepare the same day and store on wet ice or at 4°C before use.

a All wash, lysis, sorting and nuclei buffers were prepared following the guidelines from the 10x Genomics protocol. Please refer to the Demonstrated protocol for more information at the following link: https://cdn.10xgenomics.com/image/upload/v1745274143/support-documents/CG000375_DemonstratedProtocol_NucleiIsolationComplexSample_ATAC_GEX_Sequencing_Rev_D.pdf

### Buffers for nuclei isolation

**CRITICAL:** Keep the buffers on wet ice before adding to the tissue or nuclei.

#### Tissue harvesting

Hearts were harvested from C57BL/6JRj 9 weeks old male mice under deep anesthesia with 3% isoflurane. Following excision, both atria and right ventricle were removed and the left ventricle was rinsed in physiological saline solution and divided into two sagittal sections, gently blotted dry, placed in a 2 mL Eppendorf tube and immediately snap-frozen in liquid nitrogen.

#### Hardware preparation

The single-nucleus RNA-seq and single-nucleus ATAC-seq Fastq data processing have been conducted on a MacOS computer. 16 GB RAM should be sufficient for the initial analysis.

#### Data analysis section

Cell Ranger Arc pipelines were used to process the Chromium Single Cell Multiome ATAC + Gene Expression sequencing. Cell Ranger ARC (v2.0.2.) was used against the mm10-2020-A. R software packages Seurat (v5.2.0) and Signac (v1.16.0) are essential for analyzing, interpreting, and exploring single-nucleus Multiomic datasets. For this protocol, we used R version 4.3.3.

## Step-by-step method details

### Preparation of equipment and solutions

**Timing: 1 h**

Prepare all fresh solutions on the day of the experiment and keep them on wet ice throughout the procedure to preserve nuclei integrity and minimize degradation.

**CRITICAL:** Prepare fresh buffers before use and keep the buffers on wet ice before adding to the tissue/nuclei. All the tubes containing tissue and nuclei must be always on wet ice. Autoclave and treat all equipment with RNaseZap to reduce contamination. RNAse can greatly reduce nuclei yield and contribute to degradation artifacts within the cDNA. All surfaces and equipment should be decontaminated, and reagents must stay cold throughout the procedure to reduce the activity of RNAses/DNAses.

1. Prepare the following solutions fresh on the day of the experiment and keep them on wet ice throughout the procedure: Homogenization buffer 01-04, Centrifuge buffer 01, Sorting buffer, 1x Lysis Buffer, Lysis Dilution Buffer, 0.1× Lysis Buffer, Wash Buffer, and Diluted Nuclei Buffer.
2. Prepare the following items and place them on ice:
  a. A 50 mL gentleMACS C Tube containing 2.5 mL of Homogenization buffer 02.
  b. Two 15 mL Protein LoBind centrifuge tubes containing 2 mL Centrifuge buffer 01.
  c. Two empty 15 mL Protein LoBind centrifuge tubes.

### Tissue dissociation

**Timing: 15 min**

This section describes mechanical dissociation of snap-frozen murine left ventricle tissue and sequential filtration to obtain a crude nuclei suspension for downstream debris removal.

3. Remove one whole mouse snap frozen left ventricle from − 80 °C freezer storage and transfer it into a 50 mL gentleMACS C Tube containing 2.5 mL of Homogenization buffer 02.
4. Homogenize the tissue using a gentleMACS Dissociator.
  a. Select the dissociation protocol “m_mito_tissue_01”
  b. Run the protocol two consecutive times for 1 minute each.
5. Add 2.5 mL of Homogenization buffer 03 directly to the solution containing the homogenized tissue in the 50 mL gentleMACS C Tube.
6. Filter the solution sequentially through a 70 μm strainer and then through a 30 μm strainer into a 15 mL Protein LoBind centrifuge tube.
7. Wash the 30 μm strainer with 2.5 mL of Homogenization buffer 04 and let it pass through.
8. Centrifuge the 15 mL tube at 500 × *g* for 5 min in a benchtop centrifuge equipped with a swinging-bucket rotor and precooled to 4 °C.

**CRITICAL:** Use only swinging-bucket rotor for all centrifugation steps. Use of any other type of rotor is not recommended. All the centrifugation steps have been performed on a centrifuge equipped with a swinging-bucket rotor.

### Debris removal

**Timing: 45 min**

This section enriches nuclei and removes cellular debris by repeated cushion-based centrifugation, followed by fluorescence-activated nuclei sorting.

9. Remove the 15 mL tube from the centrifuge and place on ice.
  a. Discard the supernatant and resuspend the nuclei pellet in 2 mL Centrifuge buffer 01.
10. Overlay the nuclei suspension on a 2 mL cushion of Centrifuge buffer 01 contained in a 15 mL Protein LoBind centrifuge tube.

**CRITICAL:** Overlay the nuclei suspension on the Centrifuge buffer 01 cushion with care to ensure an interphase is maintained between the two.

11. Centrifuge the tube at 500 × *g* for 5 min in a benchtop centrifuge equipped with a swinging-bucket rotor and precooled to 4 °C.
12. Remove the 15 mL tube from the centrifuge and place on ice.
  a. Discard the supernatant and resuspend the nuclei pellet in 2 mL Centrifuge buffer 01.
13. Overlay the nuclei suspension on a 2 mL cushion of Centrifuge buffer 01 contained in a 15 mL Protein LoBind centrifuge tube.
14. Centrifuge the 15 mL tube at 500 × *g* for 5 min in a benchtop centrifuge equipped with a swinging-bucket rotor and precooled to 4 °C.
15. Carefully remove the tube from the centrifuge and place on ice.
  a. Discard the supernatant and add slowly on the nuclei pellet 1 mL sorting buffer without disturbing the pellet and let it incubate on ice for 5 min.

**CRITICAL:** Do not mix and disturb the pellet.

16. Mix 10 times and dissolve the nuclei pellet.
17. Centrifuge the tube at 500 × *g* for 5 min in a benchtop centrifuge equipped with a swinging-bucket rotor and precooled to 4 °C.
18. Remove the 15 mL tube from the centrifuge and place on ice.
  a. Discard the supernatant and resuspend the nuclei pellet in 1 mL sorting buffer.
19. Add 10 μL 7AAD ready-made solution to the 1 mL sorting buffer containing the nuclei and mix 10 times.
20. Incubate for minimum 5 min on ice.
21. Sort 100,000 7-AAD-positive nuclei using a BD FACSMelody (or equivalent) sorter.
  a. Follow the gating strategy described in Figure 2.
  b. Collect nuclei into a 1.5 mL Eppendorf DNA LoBind tube containing 200 μL 10% BSA and 50 μL RNase inhibitor (40 U/μL).

**CRITICAL:** Assistance is required to operate the FACS machine except for experienced users. Addition of BSA improves spinning down of nuclei after FACS sorting and will improve the final yield of nuclei recovered.

22. Remove the tube containing the sorted nuclei, mix gently 10 times and place it on ice.

### Nuclei permeabilization

**Timing: 15 min**

This section permeabilizes sorted nuclei and prepares them for downstream multiome library preparation.

23. Centrifuge the 1.5 mL tube at 500 × *g* for 5 min in a benchtop centrifuge equipped with a swinging-bucket rotor and precooled to 4 °C.
24. Remove the 1.5 mL tube from the centrifuge and place on ice.
  a. Discard the supernatant and resuspend the nuclei pellet in 100 μL 0.1× Lysis Buffer.
  b. Pipette mix gently 5–7 times until the pellet has been dissolved.
25. Incubate for exactly 2 min on ice.
26. Add 1 mL Wash Buffer and pipette mix 5–8 times.
27. Centrifuge the 1.5 mL tube at 500 × *g* for 5 min in a benchtop centrifuge equipped with a swinging-bucket rotor and precooled to 4 °C.
28. Carefully remove the 1.5 mL tube from the centrifuge and place on ice.
  a. Discard the supernatant and resuspend the nuclei pellet in 12 μL of 1× Diluted Nuclei Buffer.
  b. Mix gently until the pellet has been dissolved.

### Nuclei counting

**Timing: 15 min**

This section determines nuclei concentration and assesses nuclei quality, membrane integrity, and aggregate formation before barcoding.

29. Vortex the 0.4% trypan blue stain and centrifuge briefly.
  a. Filter through a 0.1μm mesh to remove any stain precipitate.
  b. Place 5μL in a 0.5 mL tube.
30. Mix 5μL of nuclei solution with 5μL of trypan blue stain and pipet into a disposable Countess chamber slide.

***Note:*** Important to have the trypan blue stain filtered through a 0.1μm mesh to remove any stain precipitate.

31. Determine the nuclei concentration using a Countess III FL Automated Cell Counter.

***Note:*** The nuclei concentration in these experiments ranged from 4000 to 8000 nuclei/μL.

32. Use the disposable Countess chamber slide under a brightfield microscope to check for nuclei aggregates presence as well as visually inspecting the nuclei membrane.
  a. Refer to Figure 4 for optimal and suboptimal nuclear isolation and lysis end result.

**Figure 4:**
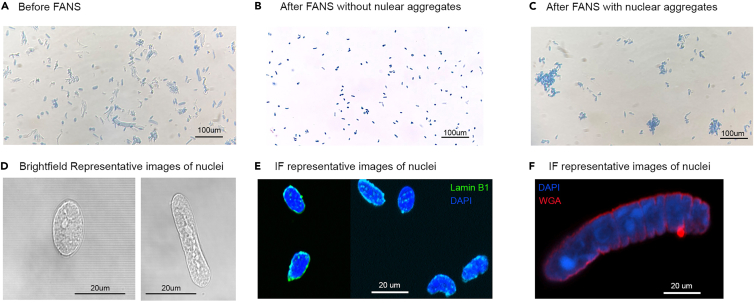
Assessment of nuclear membrane during single-nuclei isolation (A) Representative trypan blue staining of nuclei suspension prior to FANS. (B) Representative trypan blue staining of nuclei suspension after FANS, showing good separation between nuclei and absence of nuclei aggregates. (C) Example of suboptimal nuclei isolation resulting in large nuclear aggregates, shown by trypan blue staining. (D) Representative brightfield images of isolated nuclei. (E) Representative images of fluorescence microscopy images of nuclei tagged with anti-LaminB1(green) and DAPI(blue). (F) Representative images of fluorescence microscopy images of nuclei tagged with WGA(red) and DAPI(blue).
33. Proceed further with barcoding according to 10x Genomics. Chromium Next GEM Single Cell Multiome ATAC + Gene Expression Reagent Kits User Guide.^14^

### Single-nuclei quality control and library preparation

**Timing: 2 days**

This section describes preparation of single-nucleus multiome libraries and the initial processing of sequencing output.

**Figure 5:**
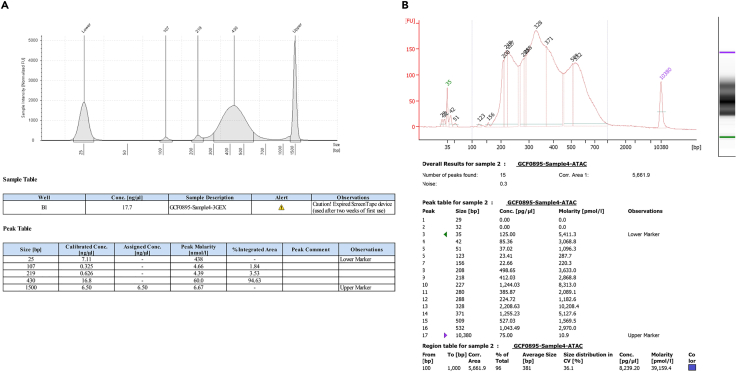
Examples of library pool QC traces (A and B) Shown are the representative traces of successful QC analyses for snRNAseq, at the cDNA amplification step (A), and snATACseq trace (B).

**Pause point:** After library preparation there is the option for a pause point. Libraries can be stored at −20°C for several months before sequencing. Representative examples of RNA and ATAC libraries can be found in Figure 5.

34. Use the Cell Ranger ARC pipeline to process the sequencing data. The sequencing statistics produced by CellRanger can be found in Table 1.

**Table 1:** Sequencing statistics from the web summary htmL report produced by CellRanger

	Sample 1	Sample 2	Sample 3	Sample 4
	RNA			
Sequenced read pairs	345,608,119	326,256,548	329,223,104	299,812,816
Mean raw reads per nucleus	42,805	44,509	34,783	42,959
Median UMI count per nucleus	226	262	200	175
Median number of genes per nucleus	194	231	174	150
Fraction of transcriptomic reads in nuclei	94.6%	94.4%	93.9%	94.1%

	**ATAC**			
Sequenced read pairs	286,883,698	368,982,509	333,410,174	327,546,041
Mean raw reads per nucleus	35,531	50,338	35,225	46,933
Number of peaks	108,495	114,233	107,326	112,153
Median high-quality fragments per nucleus	17,373	19,425	9,314	14,025
TSS enrichment score	6.65	6.55	6.38	6.83

### Single-nucleus RNA- and ATAC-seq QC per sample

**Timing: 6 h**

This section describes per-sample quality control analysis of snRNA-seq and snATAC-seq data in R before sample integration and downstream analyses.

**CRITICAL:** Before starting the analysis, confirm that you have a working R environment with all the required packages installed. Please refer to Seurat and Signac for the essential packages and related packages installation.

35. Prepare the R libraries that will be used.

>library(dplyr)

>library(scDblFinder)

>library(patchwork)

> library(ggplot2)

> library(Signac)

> library(EnsDb.Mmusculus.v79)

>library(Seurat)

>library(Azimuth)

>library(SeuratData)

>library(chromVAR)

>library(JASPAR2020)

>library(TFBSTools)

>library(motifmatchr)

>library(BSgenome.Mmusculus.UCSC.mm10)

>library(harmony)

36. Load the datasets using the Seurat Package and create the Seurat Object that contains both RNA and ATAC seq results.

>h5_file <-"//User/NameofSample/outs/filtered_feature_bc_matrix.h5"

>dataset <- Read10X_h5(h5_file, use.names = TRUE, unique.features = TRUE)

>rna_counts <- dataset$`Gene Expression`

> atac_counts <- dataset$Peaks

> SeuratObj <- CreateSeuratObject(counts = rna_counts)

> SeuratObj[["percent.mt"]] <- PercentageFeatureSet(SeuratObj, pattern = "ˆmt-")

>SeuratObj[["percent.ribo"]] <- PercentageFeatureSet(SeuratObj, pattern = "ˆRp[sl]")

>grange.counts <- StringToGRanges(rownames(atac_counts), sep = c(":", "-"))

>grange.use <- seqnames(grange.counts) %in%standardChromosomes(grange.counts)

>atac_counts <- atac_counts[as.vector(grange.use), ]

>annotations <- GetGRangesFromEnsDb(ensdb = EnsDb.Mmusculus.v79)

>seqlevelsStyle(annotations) <- 'UCSC'

>genome(annotations) <- "mm10"

>frag.file <- "//User/NameofSample/outs/atac_fragments.tsv.gz"

> chrom_assay <- CreateChromatinAssay(counts = atac_counts, sep = c(":", "-"),genome = 'mm10', fragments = frag.file, min.cells = 10, annotation = annotations)

>SeuratObj[["ATAC"]] <- chrom_assay

>DefaultAssay(SeuratObj) <- "ATAC"

>SeuratObj <- NucleosomeSignal(SeuratObj)

>SeuratObj <- TSSEnrichment(SeuratObj)

37. Perform the quality control per nucleus based on the parameters described to Table 2.

**Table 2:** Quality control thresholds and exclusion criteria applied to each sample

RNA	QC parameters
nCount_RNA	Lowest cut off values between 100 and 75
nFeature_RNA	Lowest cut off values between 100 and 65 and highest cut off values between 700 and 2443
percent.mt	Lowest cut off values between 3 and 5

ATAC	QC parameters
nCount	Lowest cut off values between 1000 and 2000
TSS.enrichment	Lowest cut off values between 2.8 and 3.3
scDblFinder	Singlet

>nCount_RNA_lower <- Cutoff_Value_per_sample

>nFeature_RNA_lower <- Cutoff_Value_per_sample

>nFeature_RNA_upper <- Cutoff_Value_per_sample

>percent.mt_upper <- Cutoff_Value_per_sample

>nCount_ATAC_lower <- Cutoff_Value_per_sample

>TSS.enrichment_lower <- Cutoff_Value_per_sample

>doublets <- scDblFinder(sce = as.matrix(SeuratObj@assays$RNA$counts))

>SeuratObj$doublet_score <- doublets$scDblFinder.score

>SeuratObj$doublet <- doublets$scDblFinder.class

>SeuratObj.filt <- subset(SeuratObj,subset =nCount_RNA > nCount_RNA_lower &nFeature_RNA > nFeature_RNA_lower &nFeature_RNA < nFeature_RNA_upper &percent.mt < percent.mt_upper &nCount_ATAC > nCount_ATAC_lower &TSS.enrichment > TSS.enrichment_lower &doublet == "singlet")

38. Perform modality-specific preprocessing of the RNA and ATAC data by applying SCTransform, PCA, and RNA UMAP, followed by TF-IDF normalization, SVD, and ATAC UMAP.

**CRITICAL:** For RNA preprocessing the number of principal components retained for downstream analysis was selected based on inspection of elbow plots. For ATAC preprocessing the number of latent semantic indexing (LSI) dimensions retained was selected based on the variance structure of the components and exclusion of non-informative dimensions like the first LSI component which is often dominated by technical variation, especially sequencing depth / library size rather than biologically meaningful chromatin accessibility differences.

>DefaultAssay(SeuratObj.filt) <- "RNA"

>SeuratObj.filt <- SCTransform(SeuratObj.filt, return.only.var.genes = FALSE, verbose = FALSE) %>%

RunPCA() %>%

RunUMAP(dims = 1:50, reduction.name = 'umap.rna', reduction.key = 'rnaUMAP_')

>DefaultAssay(SeuratObj.filt) <- "ATAC"

>SeuratObj.filt <- RunTFIDF(SeuratObj.filt)

>SeuratObj.filt <- FindTopFeatures(SeuratObj.filt, min.cutoff = 'q0')

>SeuratObj.filt <- RunSVD(SeuratObj.filt)

>SeuratObj.filt <- RunUMAP(SeuratObj.filt, reduction = 'lsi', dims = 2:50, reduction.name = "umap.atac", reduction.key = "atacUMAP_")

>SeuratObj.filt <- FindMultiModalNeighbors(SeuratObj.filt, reduction.list = list("pca", "lsi"), dims.list = list(1:50, 2:50))

>SeuratObj.filt <- RunUMAP(SeuratObj.filt, nn.name = "weighted.nn", reduction.name = "wnn.umap", reduction.key = "wnnUMAP_")

39. Annotate the nuclei to respective cell types for each sample.

>DefaultAssay(SeuratObj.filt) <- "RNA"

>SeuratObj.heart <- RunAzimuth(SeuratObj.filt, reference = "heartref")

>SeuratObj.filt <- AddMetaData(SeuratObj.filt, SeuratObj.heart$predicted.celltype.l1, col.name = "predicted.celltype.l1")

>SeuratObj.filt <- AddMetaData(SeuratObj.filt, SeuratObj.heart$predicted.celltype.l2, col.name = "predicted.celltype.l2")

>SeuratObj.filt <- AddMetaData(SeuratObj.filt, SeuratObj.heart$predicted.celltype.l1.score, col.name = "predicted.celltype.l1.score")

>SeuratObj.filt <- AddMetaData(SeuratObj.filt, SeuratObj.heart$predicted.celltype.l2.score, col.name = "predicted.celltype.l2.score")

### Merging of all samples and downstream analysis

**Timing: 4 h**

This section describes integration of all individual sample objects and the downstream analyses used to generate the final multimodal clustering and visualization results.

40. Merge the Seurat objects of each sample.

>rds.files<-list.files("//UserAllSamples/data", full.names = TRUE)

>SeuratObj.filt_Sample1<- readRDS(rds.files[1])

>SeuratObj.filt_Sample2<- readRDS(rds.files[2])

>SeuratObj.filt_Sample3<- readRDS(rds.files[3])

>SeuratObj.filt_Sample4<- readRDS(rds.files[4])

>Fragments(SeuratObj.filt_Sample1@assays$ATAC) <- NULL

>Fragments(SeuratObj.filt_Sample2@assays$ATAC) <- NULL

>Fragments(SeuratObj.filt_Sample3@assays$ATAC) <- NULL

>Fragments(SeuratObj.filt_Sample4@assays$ATAC) <- NULL

>fragments1 <- CreateFragmentObject(path = "// User/NameofSample/outs/atac_fragments.tsv.gz", cells = colnames(SeuratObj.filt_Sample1), validate.fragments = TRUE)

>fragments2 <- CreateFragmentObject(path = "// User/NameofSample/outs/atac_fragments.tsv.gz", cells = colnames(SeuratObj.filt_Sample2), validate.fragments = TRUE)

>fragments3 <- CreateFragmentObject(path = "// User/NameofSample/outs/atac_fragments.tsv.gz", cells = colnames(SeuratObj.filt_Sample3), validate.fragments = TRUE)

>fragments4 <- CreateFragmentObject(path = "// User/NameofSample/outs/atac_fragments.tsv.gz", cells = colnames(SeuratObj.filt_Sample4), validate.fragments = TRUE)

>Fragments(SeuratObj.filt_Sample1@assays$ATAC) <- fragments1

>Fragments(SeuratObj.filt_Sample2@assays$ATAC) <- fragments2

>Fragments(SeuratObj.filt_Sample3@assays$ATAC) <- fragments3

>Fragments(SeuratObj.filt_Sample4@assays$ATAC) <- fragments4

>DefaultAssay(SeuratObj.filt_Sample1) <- "RNA"; DefaultAssay(SeuratObj.filt_Sample2) <- "RNA"; DefaultAssay(SeuratObj.filt_Sample3) <- "RNA";DefaultAssay(SeuratObj.filt_Sample4) <- "RNA"

> SeuratObj.all <- merge(x = SeuratObj.filt_Sample1, y = list(SeuratObj.filt_Sample2, SeuratObj.filt_Sample3, SeuratObj.filt_Sample4))

41. Perform clustering on the integrated multimodal dataset and generate UMAP visualizations for RNA, ATAC, and WNN embeddings and visualize the integrated dataset.

**CRITICAL:** For RNA preprocessing the number of principal components retained for downstream analysis was selected based on inspection of elbow plots. For ATAC preprocessing the number of latent semantic indexing (LSI) dimensions retained was selected based on the variance structure of the components and exclusion of non-informative dimensions like the first LSI component which is often dominated by technical variation, especially sequencing depth / library size rather than biologically meaningful chromatin accessibility differences.

>DefaultAssay(SeuratObj.all) <- "RNA"

>SeuratObj.all <- SCTransform(SeuratObj.all, return.only.var.genes = FALSE)

>SeuratObj.all <- RunPCA(SeuratObj.all, npcs = 100, verbose = F)

>DefaultAssay(SeuratObj.all) <- "ATAC"

>SeuratObj.all <- RunTFIDF(SeuratObj.all)

>SeuratObj.all <- FindTopFeatures(SeuratObj.all, min.cutoff = 'q0')

>SeuratObj.all <- RunSVD(SeuratObj.all, n = 100)

>DefaultAssay(SeuratObj.all) <- "SCT"

>SeuratObj.all <- IntegrateLayers( object = SeuratObj.all, method = HarmonyIntegration,

orig.reduction = "pca", new.reduction = "harmony",

normalization.method = "SCT", verbose = FALSE)

>SeuratObj.all <- FindMultiModalNeighbors(SeuratObj.all, reduction.list = list("harmony", "lsi"), dims.list = list(1:100, 2:100))

>SeuratObj.all <- RunUMAP(SeuratObj.all, nn.name = "weighted.nn", reduction.name = "wnn.harmony.umap", reduction.key = "wnnUMAP_")

>SeuratObj.all[["RNA"]] <- JoinLayers(SeuratObj.all[["RNA"]])

42. Add motif annotations to the ATAC assay and compute motif accessibility scores using chromVAR.

>DefaultAssay(SeuratObj.all) <- "ATAC"

> pwm_set <- getMatrixSet(x = JASPAR2020, opts = list(species = 10090, all_versions = FALSE))

>motif.matrix <- CreateMotifMatrix(features = granges(SeuratObj.all), pwm = pwm_set, genome = 'mm10', use.counts = FALSE)

>motif.object <- CreateMotifObject(data = motif.matrix, pwm = pwm_set)

>SeuratObj.all <- SetAssayData(SeuratObj.all, assay = 'ATAC', layer = 'motifs', new.data = motif.object)

>SeuratObj.all <- RunChromVAR( object = SeuratObj.all, genome = BSgenome.Mmusculus.UCSC.mm10 )

43. Annotate ATAC peaks with sequence statistics and infer regulatory peak-to-gene associations.

>DefaultAssay(SeuratObj.all) <- "ATAC"

> SeuratObj.all <- RegionStats(SeuratObj.all, genome = BSgenome.Mmusculus.UCSC.mm10)

>SeuratObj.all <- LinkPeaks( object = SeuratObj.all,

peak.assay = "ATAC",

expression.assay = "SCT"

)

## Expected outcomes

**Figure 6:**
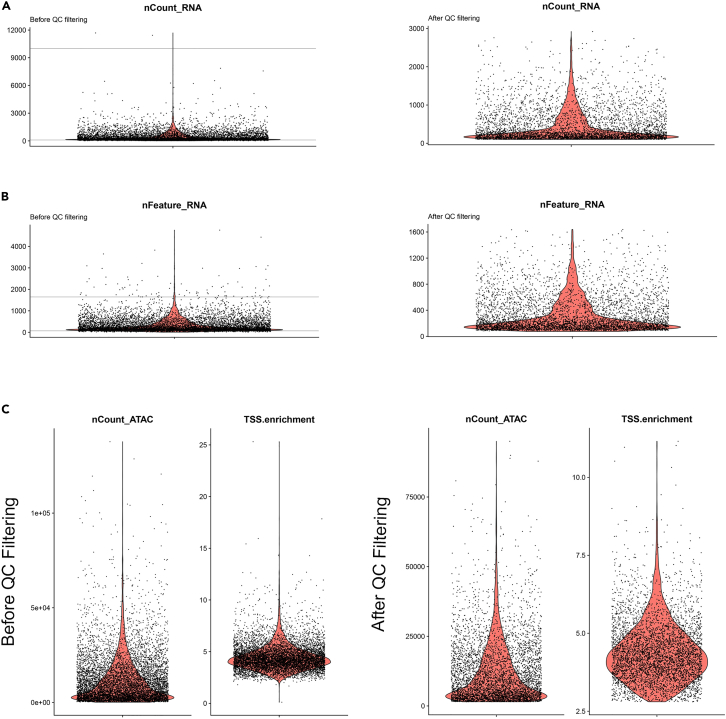
Quality control assessment of snRNA-seq and snATAC-seq data (A) Violin plots showing the distribution of per-nucleus RNA nCount values before filtering and after removal of low-quality nuclei. (B) Violin plots showing the distribution of per-nucleus RNA nFeature values before filtering and after removal of low-quality nuclei. (C) Violin plots showing the distribution of per-nucleus ATAC nCount values and TSS enrichment scores before filtering and after removal of low-quality nuclei.

**Figure 7:**
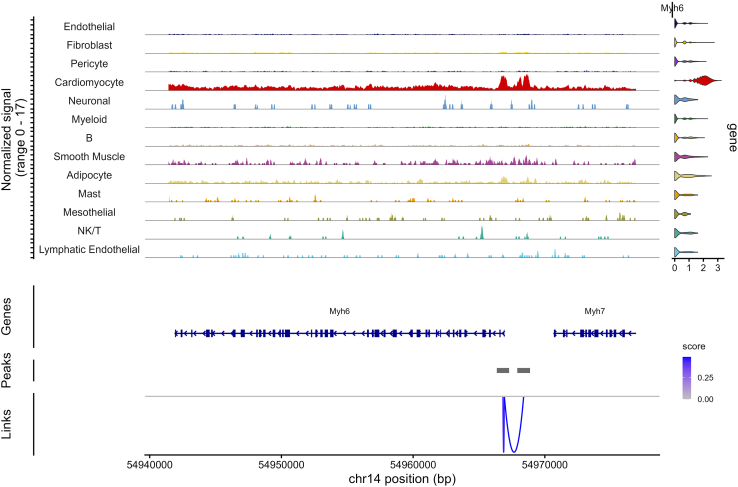
Visualization of cluster-specific genomic regions Pseudobulk chromatin accessibility tracks at the *Myh6* locus, with peak-to-gene linkage analysis showing correlations between accessible chromatin regions (peaks) and expression of nearby genes.

**Figure 8:**
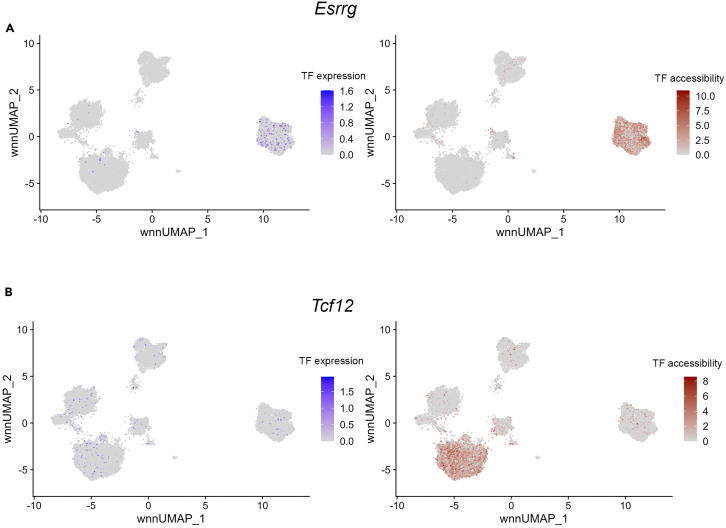
Cell-type specific transcription factors colored by RNA expression (left) and motif accesibility (right) Representative UMAP visualization showing unique transcription factors for the cardiomyocytes (A) and fibroblasts (B), based on combined RNA expression and ATAC motif accessibility analyses. Cells are colored according to normalized RNA expression levels (left) and corresponding motif accessibility scores (right).

Single-nucleus multiomics is an emerging field with limited standardized protocols for nuclei isolation from fibrous tissue like the heart. Here we propose a protocol that results in the isolation of single-nuclei solutions from post mortem murine cardiac left ventricle. This was achieved in our protocol through standardized mechanical dissociation, sequential filtration steps combined with FANS, which efficiently removed the vast majority of debris. A BD FACSMelody sorter was used with a nozzle size of 100 μm. Here cardiac nuclei are identified by forward scatter (FSC) and side scatter (SSC). Fluorescent gating allowed the separation of nuclei (7AAD-positive) and non-nuclei debri (7AAD-negative) from cardiac tissue. A third gate identified single nuclei by FSC-Height and FSC-Width pulse (Figure 2). The effect of FANS for debri removal is evident in Figure 4 which illustrates representative trypan blue staining images showing the nuclei solution before FANS (Figure 4A), after FANS (Figure 4B) showing that FANS is vital for capturing nuclei populations and removing all unstained debri. During the protocol, at step 32 we evaluate the presence of nuclei aggregates in the single nuclei solution (Figure 4C) under light microscopy after the use of trypan blue staining. The library preparation for RNA and ATAC took typically 2 days to complete including library quality control(Figure 5). After sequencing, a necessary quality control evaluation of snRNA and snATAC-seq data was performed in a per sample base removing of low quality nuclei(Figure 6). After filtration and merging of all the samples, integrated and modality-specific clustering generated UMAP projections of the snRNA-seq, snATAC-seq, and WNN-integrated clustering where each spot represents a cell type annotated nucleus(Figure 3A). This workflow produced an overall cell-type composition consistent with previous reports in the literature (Figure 3B) with with only minor variation between samples(Figure 3C), demonstrating that the protocol is robust and well suited for state-of-the-art cardiac research.^4^^,^^6^^,^^17^ To further illustrate the integrative power of this workflow, we examined chromatin accessibility and gene expression at the *Myh6* locus, a typical marker of cardiomyocytes where there is a noticeable difference on both gene expression and chromatin accessibility between cardiomyocytes and other cell types. Pseudobulk peak-to-gene linkage analysis revealed strong associations between regulatory elements and *Myh6* expression (Figure 7). This highlights the ability of combined snATAC- and snRNA-seq to pinpoint specific regulatory interactions at functionally important cardiac genes, providing direct evidence of transcriptional regulation embedded in chromatin structure. To further validate cell type annotation and demonstrate unique cell type chromatin accessibility, representative transcription factors were examined across major cardiac cell populations. As shown in Figure 8 cardiomyocytes and fibroblasts display cell type–specific patterns of both RNA expression and motif accessibility of selected transcription factors.

## Limitations

Although the workflow is designed to produce highly concentrated, single-nuclei suspension, certain limitations should be acknowledged. The requirement for specialized equipment, including the gentleMACS Dissociator and a fluorescence-activated nuclei sorter, increases cost and may limit accessibility in resource-constrained settings. Furthermore, although low-pressure sorting conditions are used, FANS-based nuclei purification can still impose hydraulic stress that may subtly perturb nuclear membranes. Moreover the multiple washing and low-speed centrifugation steps incorporated to improve cleanup and reduce ambient contamination may increase the risk of material loss, particularly when starting from small quantities of tissue. Similarly, while density-based purification improves sample quality, we cannot exclude the possibility that the sucrose cushion gradient introduces subtle biases in the spectrum of nuclei recovered. This potential bias has not been systematically evaluated across tissue types and may require empirical validation depending on the biological context. Finally, while the protocol performs robustly in murine ventricular tissue, it has not yet been systematically validated in human cardiac samples, and its performance in human patient-derived myocardium remains to be established. In addition, the protocol has not yet been evaluated on tissue that has been stored frozen for extended periods, such as archival samples from biobanks, and its performance on long-term cryopreserved material therefore remains to be determined.

## Troubleshooting

### Problem 1

Low nuclei recovery. (related to Step 21).

Low nuclei recovery can occur for several reasons during sample processing. Recovery may be too low when the number of sorted nuclei is significantly below 100,000, making pellet formation difficult to detect after centrifugation. The type of centrifuge rotor used can also influence pellet visibility, as fixed-angle rotors may not support pellet formation as effectively as swinging-bucket rotors. Furthermore, centrifugation times that are too short may be insufficient to allow proper nuclei pelleting. These factors can reduce sample quality and complicate downstream analyses. To ensure reliable and reproducible results, consider the following potential strategies.

### Potential solution

• **Aim for sufficient nuclei numbers:** Try to isolate at least 100,000 nuclei per sample, as lower numbers may lead to poor recovery and difficulty detecting a visible pellet.
• **Use an appropriate centrifuge rotor:** A swinging-bucket rotor centrifuge should be used for pelleting nuclei, as it provides more consistent pellet formation than a fixed-angle rotor.
• **Improve pellet visibility and centrifugation conditions:** The use of 7-AAD stain can help by giving the nuclei suspension a distinct red color, which makes pellet identification easier. In our experience, centrifugation for 5 min was sufficient to form a visible pellet when 100,000 nuclei were sorted and a swinging-bucket rotor was used. In contrast, no visible pellet was observed with a fixed-angle rotor, even after 20 min of centrifugation at 500 × g.

### Problem 2

Presence of many nuclei aggregates (related to Step 32).

The appearance of nuclei clumps during sample preparation may indicate that one or more steps in the protocol are not optimal. Suboptimal BSA concentration in the buffers can reduce nuclei stability and increase the likelihood of aggregates forming. The FANS sorting instrument itself may also contribute to this issue, as machines operating at high hydraulic pressure can produce more variable results and may increase nuclei membrane stress during sorting. The presence of nuclei clumps can negatively affect sample quality, reduce sorting efficiency, and compromise downstream analyses. To improve consistency and minimize nuclei aggregation, consider the following potential strategies.

### Potential solution

• **Use a gentler dissociation program:** Select a milder tissue dissociation setting to better preserve nuclear membrane integrity. The gentleMACS dissociator offers a range of programs for both rodent and human tissues that can be tested during optimization.
• **Optimize BSA concentration in buffers:** Maintain BSA concentration between 1 and 2%, as lower concentrations increase the chance of nuclei aggregation.
• **Use a low-pressure nuclei sorter:** A low hydraulic pressure sorter, such as the FACSMelody or an equivalent system, may provide more consistent results than higher-pressure sorters such as the FACSAria.
• **Identify the source of the problem during optimization:** During trial runs, examine samples under a brightfield microscope after each major step to determine where nuclei aggregates first begin to appear.

## Resource availability

### Lead contact

Further information and requests for resources and reagents should be directed to and will be fulfilled by the lead contact, Jan Magnus Aronsen (j.m.aronsen@medisin.uio.no).

### Technical contact

Questions about the technical specifics of performing the protocol should be directed to and will be fulfilled by the technical contact, Ioanni Veras (ioanni.veras@medisin.uio.no).

### Materials availability

This study did not generate new unique reagents or materials.

### Data and code availability

Code used in this analysis is available in the supplementary document included in this article. Any data will be made available upon request.

## Acknowledgments

We gratefully acknowledge funding from the Research Council of Norway (grant no. 325192). We thank the Genomics Core Facility at 10.13039/100031365Oslo University Hospital for providing sequencing services for this project, the Bioinformatics Core Facility at 10.13039/100031365Oslo University Hospital for providing bioinformatics support, and the Advanced Light Microscopy Core Facility at 10.13039/100031365Oslo University Hospital for providing microscopy services. Moreover, we thank the Flow Cytometry Core Facility (FCCF) at Oslo University Hospital for access to equipment and assistance with sorting. The graphical abstract was created with BioRender.com.

## Author contributions

I.V.: conceptualization, methodology, investigation, visualization, and writing – original draft. O.S.E.: data acquisition and visualization and writing – review and editing. F.O.L. and A.O.M.: writing – review and editing and supervision. J.M.A.: conceptualization, supervision, project administration, funding acquisition, and writing – review and editing.

## Declaration of interests

The authors declare no competing interests.

## Declaration of generative AI and AI-assisted technologies in the writing process

During the preparation of this work, the authors used ChatGPT (OpenAI) to assist with language editing and improvement of readability. The authors reviewed and edited the content as needed and take full responsibility for the content of the publication.
