# Supplementary material for: Protocol for isolation of nuclei from murine cardiac tissue for single-nucleus multiomic sequencing
Source: STAR Protoc. 2026 Jun 6;7(2):104615. doi: 10.1016/j.xpro.2026.104615 (PMC13260199; doi:10.1016/j.xpro.2026.104615)
Supplement: Document S1. Supplemental material [file mmc1.pdf]

SUPPLEMENTAL MATERIAL

Code used in this article

```
library(dplyr)
library(scDblFinder)
library(patchwork)
library(ggplot2)
library(Signac)
library(EnsDb.Mmusculus.v79)
library(Seurat)
library(Azimuth)
library(SeuratData)
library(chromVAR)
library(JASPAR2020)
library(TFBSTools)
library(motifmatchr)
library(BSgenome.Mmusculus.UCSC.mm10)

#This part starts the per sample analysis#

h5_file <-"//User/NameofSample/outs/filtered_feature_bc_matrix.h5"
dataset <- Read10X_h5(h5_file, use.names = TRUE, unique.features = TRUE)
rna_counts <- dataset$`Gene Expression`
atac_counts <- dataset$Peaks
SeuratObj <- CreateSeuratObject(counts = rna_counts)
SeuratObj[["percent.mt"]] <- PercentageFeatureSet(SeuratObj, pattern =
"^mt-")
SeuratObj[["percent.ribo"]] <- PercentageFeatureSet(SeuratObj, pattern =
"^Rp[sl]")
grange.counts <- StringToGRanges(rownames(atac_counts), sep = c(":", "-"))
grange.use <- seqnames(grange.counts)
%in%standardChromosomes(grange.counts)
atac_counts <- atac_counts[as.vector(grange.use), ]
annotations <- GetGRangesFromEnsDb(ensdb = EnsDb.Mmusculus.v79)
seqlevelsStyle(annotations) <- 'UCSC'
genome(annotations) <- "mm10"
frag.file <- "//User/NameofSample/outs/atac_fragments.tsv.gz"
chrom_assay <- CreateChromatinAssay(counts = atac_counts, sep = c(":", "-
"),genome = 'mm10', fragments = frag.file, min.cells = 10, annotation =
annotations)
SeuratObj[["ATAC"]] <- chrom_assay
DefaultAssay(SeuratObj) <- "ATAC"
SeuratObj <- NucleosomeSignal(SeuratObj)
nCount_RNA_lower <- Cutoff_Value_per_sample #Sample 1: 100, Sample 2:
100,Sample 3:75 , Sample 4: 100
nFeature_RNA_lower <- Cutoff_Value_per_sample #Sample 1: 70, Sample 2:100
,Sample 3: 65, Sample 4:100
nFeature_RNA_upper <- Cutoff_Value_per_sample#Sample 1: 1647, Sample 2:
1235,Sample 3: 700, Sample 4:2443
percent.mt_upper <- Cutoff_Value_per_sample#Sample 1: 3, Sample 2:3
,Sample 3: 3.3, Sample 4:5
```

```
nCount_ATAC_lower <- Cutoff_Value_per_sample#Sample 1: 1500, Sample 2:
1000,Sample 3:1600 , Sample 4:2000
TSS.enrichment_lower <- Cutoff_Value_per_sample#Sample 1: 2.8, Sample 2:3
,Sample 3: 3.3, Sample 4:3
doublets <- scDblFinder(sce = as.matrix(SeuratObj@assays$RNA$counts))
SeuratObj$doublet_score <- doublets$scDblFinder.score
SeuratObj$doublet <- doublets$scDblFinder.class
DefaultAssay(SeuratObj.filt) <- "RNA"
SeuratObj.filt <- SCTransform(SeuratObj.filt, return.only.var.genes =
FALSE, verbose = FALSE) %>%
  RunPCA() %>%
  RunUMAP(dims = 1:50, reduction.name = 'umap.rna', reduction.key =
'rnaUMAP_')
DefaultAssay(SeuratObj.filt) <- "ATAC"
SeuratObj.filt <- RunTFIDF(SeuratObj.filt)
SeuratObj.filt <- FindTopFeatures(SeuratObj.filt, min.cutoff = 'q0')
SeuratObj.filt <- RunSVD(SeuratObj.filt)
SeuratObj.filt <- RunUMAP(SeuratObj.filt, reduction = 'lsi', dims = 2:50,
reduction.name = "umap.atac", reduction.key = "atacUMAP_")
SeuratObj.filt <- FindMultiModalNeighbors(SeuratObj.filt, reduction.list =
list("pca", "lsi"), dims.list = list(1:50, 2:50))
DefaultAssay(SeuratObj.filt) <- "RNA"
SeuratObj.heart <- RunAzimuth(SeuratObj.filt, reference = "heartref")
SeuratObj.filt <- AddMetaData(SeuratObj.filt,
SeuratObj.heart$predicted.celltype.l1, col.name = "predicted.celltype.l1")
SeuratObj.filt <- AddMetaData(SeuratObj.filt,
SeuratObj.heart$predicted.celltype.l2, col.name = "predicted.celltype.l2")
SeuratObj.filt <- AddMetaData(SeuratObj.filt,
SeuratObj.heart$predicted.celltype.l1.score, col.name =
"predicted.celltype.l1.score")
SeuratObj.filt <- AddMetaData(SeuratObj.filt,
SeuratObj.heart$predicted.celltype.l2.score, col.name =
"predicted.celltype.l2.score")
saveRDS(SeuratObj.filt, file = paste0(output.dir,"NameofSample", ".rds"))


#This part involves the merging and analysis on all 4 samples analysis#

rds.files<-list.files("//UserAllSamples/data", full.names = TRUE)
SeuratObj.filt_Sample1<- readRDS(rds.files[1])
SeuratObj.filt_Sample2<- readRDS(rds.files[2])
SeuratObj.filt_Sample3<- readRDS(rds.files[3])
SeuratObj.filt_Sample4<- readRDS(rds.files[4])
Fragments(SeuratObj.filt_Sample1@assays$ATAC) <- NULL
Fragments(SeuratObj.filt_Sample2@assays$ATAC) <- NULL
Fragments(SeuratObj.filt_Sample3@assays$ATAC) <- NULL
Fragments(SeuratObj.filt_Sample4@assays$ATAC) <- NULL
fragments1 <- CreateFragmentObject(path = "//
User/NameofSample/outs/atac_fragments.tsv.gz", cells =
colnames(SeuratObj.filt_Sample1), validate.fragments = TRUE)
fragments2 <- CreateFragmentObject(path = "//
User/NameofSample/outs/atac_fragments.tsv.gz", cells =
colnames(SeuratObj.filt_Sample2), validate.fragments = TRUE)
```

```
fragments3 <- CreateFragmentObject(path = "//
User/NameofSample/outs/atac_fragments.tsv.gz", cells =
colnames(SeuratObj.filt_Sample3), validate.fragments = TRUE)
fragments4 <- CreateFragmentObject(path = "//
User/NameofSample/outs/atac_fragments.tsv.gz", cells =
colnames(SeuratObj.filt_Sample4), validate.fragments = TRUE)
Fragments(SeuratObj.filt_Sample1@assays$ATAC) <- fragments1
Fragments(SeuratObj.filt_Sample2@assays$ATAC) <- fragments2
Fragments(SeuratObj.filt_Sample3@assays$ATAC) <- fragments3
Fragments(SeuratObj.filt_Sample4@assays$ATAC) <- fragments4
DefaultAssay(SeuratObj.filt_Sample1) <- "RNA";
DefaultAssay(SeuratObj.filt_Sample2) <- "RNA";
DefaultAssay(SeuratObj.filt_Sample3) <-
"RNA";DefaultAssay(SeuratObj.filt_Sample4) <- "RNA"
SeuratObj.all <- merge(x = SeuratObj.filt_Sample1, y =
list(SeuratObj.filt_Sample2, SeuratObj.filt_Sample3,
SeuratObj.filt_Sample4))
DefaultAssay(SeuratObj.all) <- "RNA"
SeuratObj.all <- SCTransform(SeuratObj.all, return.only.var.genes = FALSE)
SeuratObj.all <- RunPCA(SeuratObj.all, npcs = 100, verbose = F)
SeuratObjRNA <- RunUMAP(SeuratObj.all, dims = 1:50, reduction.name =
'umap.rna', reduction.key = 'rnaUMAP_')
DefaultAssay(SeuratObj.all) <- "ATAC"
SeuratObj.all <- RunTFIDF(SeuratObj.all)
SeuratObj.all <- FindTopFeatures(SeuratObj.all, min.cutoff = 'q0')
SeuratObj.all <- RunSVD(SeuratObj.all, n = 100)
SeuratObjATAC <- RunUMAP(SeuratObj.all, reduction = 'lsi', dims = 2:50,
reduction.name = "umap.atac", reduction.key = "atacUMAP_")
DefaultAssay(SeuratObj.all) <- "SCT"
SeuratObj.all <- IntegrateLayers(
  object = SeuratObj.all, method = HarmonyIntegration,
  orig.reduction = "pca", new.reduction = "harmony",
  normalization.method = "SCT", verbose = FALSE)
SeuratObj.all <- FindMultiModalNeighbors(SeuratObj.all, reduction.list =
list("harmony", "lsi"), dims.list = list(1:100, 2:100))
SeuratObj.all <- RunUMAP(SeuratObj.all, nn.name = "weighted.nn",
reduction.name = "wnn.harmony.umap", reduction.key = "wnnUMAP_")
SeuratObj.all[["RNA"]] <- JoinLayers(SeuratObj.all[["RNA"]])
DefaultAssay(SeuratObj.all) <- "ATAC"
pwm_set <- getMatrixSet(x = JASPAR2020, opts = list(species = 10090,
all_versions = FALSE))
motif.matrix <- CreateMotifMatrix(features = granges(SeuratObj.all), pwm =
pwm_set, genome = 'mm10', use.counts = FALSE)
motif.object <- CreateMotifObject(data = motif.matrix, pwm = pwm_set)
SeuratObj.all <- SetAssayData(SeuratObj.all, assay = 'ATAC', layer =
'motifs', new.data = motif.object)
SeuratObj.all <- RunChromVAR(
  object = SeuratObj.all,
  genome = BSgenome.Mmusculus.UCSC.mm10
)
DefaultAssay(SeuratObj.all) <- "ATAC"
 SeuratObj.all <- RegionStats(SeuratObj.all, genome =
BSgenome.Mmusculus.UCSC.mm10)
SeuratObj.all <- LinkPeaks(
```

```
  object = SeuratObj.all,
  peak.assay = "ATAC",
  expression.assay = "SCT"
)



#For figures:
tol20<- c(
  "NK/T" = "#44aa99",  # coral red
  "Cardiomyocyte" = "red3",  # deep red
  "Fibroblast"= "gold2",  # dark green
  "Endothelial"= "blue4",  # blue
  "Pericyte"= "blueviolet",  # brown
  "Smooth Muscle"= "#AA4499",  # magenta
  "Myeloid"= "forestgreen",  # golden
  "Lymphatic Endothelial"= "#66CCEE",  # cyan
  "Adipocyte"= "#DDCC77",  # sand
  "Mesothelial"= "#999933",  # olive
  "Mast"= "#E69F00",   # navy
  "Neuronal"= "#6699CC",   # navy
  "B" = "orange"   # navy
)

#Umap generation
p1 <- DimPlot(SeuratObjRNA, reduction = "umap.rna", group.by =
"predicted.celltype.l1",  repel = TRUE, cols = tol20,pt.size = 0.6) +
ggtitle("RNA")
p2 <- DimPlot(SeuratObjATAC, reduction = "umap.atac", group.by =
"predicted.celltype.l1", repel = TRUE, cols = tol20,pt.size = 0.6) +
ggtitle("ATAC")
p3 <- DimPlot(SeuratObj.all, reduction = "wnn.harmony.umap", group.by =
"predicted.celltype.l1", repel = TRUE, cols = tol20,pt.size = 0.6) +
ggtitle("WNN")
p1 + p2 + p3 & NoLegend() & theme(plot.title = element_text(hjust = 0.5))

#Pie chart of total Cell type percentages
celltype_counts <- table(SeuratObj.all@meta.data$predicted.celltype.l1)

celltype_df <- as.data.frame(celltype_counts)
colnames(celltype_df) <- c("CellType", "Count")
celltype_df$Percentage <- 100 * celltype_df$Count / sum(celltype_df$Count)
celltype_df$CellType <- factor(celltype_df$CellType, levels =
names(tol20))

# build labels in tol20 order
label_df <- merge(
data.frame(CellType = names(tol20)),
celltype_df[, c("CellType", "Percentage")],
by = "CellType",
all.x = TRUE,
sort = FALSE
)
```

```
label_df$Percentage[is.na(label_df$Percentage)] <- 0

legend_labels <- setNames(
paste0(label_df$CellType, " (", round(label_df$Percentage, 1), "%)"),
label_df$CellType
)

ggplot(celltype_df, aes(x = 2, y = Percentage, fill = CellType)) +
geom_bar(stat = "identity", width = 1, color = "white") +
coord_polar(theta = "y") +
xlim(0.5, 2.5) +
scale_fill_manual(values = tol20, breaks = names(tol20), labels =
legend_labels) +
theme_void() +
ggtitle("Donut Plot: Cell Type Composition")

#Bar plot for a per sample cell type percentages
plot_df <- SeuratObj.all@meta.data %>%
dplyr::count(orig.ident, predicted.celltype.l1, name = "Count") %>%
dplyr::group_by(orig.ident) %>%
dplyr::mutate(Percentage = 100 * Count / sum(Count)) %>%
dplyr::ungroup()

plot_df$predicted.celltype.l1 <- factor(
plot_df$predicted.celltype.l1,
levels = names(tol20)
)

p_bar <- ggplot(
plot_df,
aes(x = orig.ident, y = Percentage, fill = predicted.celltype.l1)
) +
geom_bar(stat = "identity", width = 0.8) +
facet_wrap(~ orig.ident, ncol = 2, scales = "fixed") +
scale_fill_manual(values = tol20, drop = FALSE) +
labs(
x = NULL,
y = "Cell type percentage (%)",
fill = "Cell type"
) +
theme_classic() +
theme(
axis.text.x = element_blank(),
axis.ticks.x = element_blank(),
strip.background = element_blank(),
strip.text = element_text(face = "bold")
)

p_bar

#Peak to genes for Myh6
gene.links <- Links(SeuratObj.all)
```

```
idents.plot <- c("NK/T", "Fibroblast","Endothelial","Pericyte", "Smooth
Muscle", "Cardiomyocyte", "Myeloid","Lymphatic Endothelial","
Adipocyte","Mesothelial","Mast")
Idents(SeuratObj.all) <- "predicted.celltype.l1"
cols_use <- tol20
p1 <- CoveragePlot(
   object = SeuratObj.all,
   region = "Myh6",
   features = "Myh6",
   expression.assay = "SCT",
   idents = idents.plot,
   extend.upstream = 500,
   extend.downstream = 10000
 ) &
scale_fill_manual(values = cols_use, drop = FALSE) &
scale_colour_manual(values = cols_use, drop = FALSE)

p1


#Specific TFs for Cardiomyocytes and Fibroblasts type

tf_gene <- "Tcf12" #Essrg

# Find motif ID corresponding to Tcf12 or Essrg
motif.name <- ConvertMotifID(SeuratObj.all, name = tf_gene)

rna_feature <- paste0("sct_", tf_gene)

gene_plot <- FeaturePlot(
SeuratObj.all,
features = rna_feature,
reduction = "wnn.harmony.umap"
)

motif_plot <- FeaturePlot(
SeuratObj.all,
features = motif.name,
min.cutoff = 0,
cols = c("lightgrey", "darkred"),
reduction = "wnn.harmony.umap"
)
tcf12_plot <- gene_plot | motif_plot
```
